# News Items

**Published:** 1885-01

**Authors:** 


### News Items.

Illinois Physicians—Register of Practitioners Holding Certificates Issued by the State Board of Health.

The Revised Official Register of physicians and midwives in practice in the State is issued in a volume of 324 pages. It contains the Medical-Practice act, and the State Board of Health account; a section on practitioners and mode of procedure under the Medical-Practice act, an official register of physicians, resident and non-resident, a list of revoked certificates, the necrological record of the year, a directory of the medical societies in the State, an official register of midwives, and an addenda bringing the record up to Dec. 1, inst.

The Register embraces the names, post-office addresses, and other data necessary for the purpose of identification of 5,585 physicians to whom certificates have been issued by the Board of Health under the act approved May 29, 1877, and who comprise the majority of those engaged in medical practice in the State. To this number are to be added 300 practitioners who are exempt from the provisions of the act by reason of having practiced ten years in the State prior to July 1, 1877, so that the whole number of practitioners in the State at the close of this year is 5,885. Of the 5,585 certificated practitioners there are 4,780 who hold certificates from the board based upon satisfactory proof of having received diplomas or licenses from legally-chartered medical institutions in good standing; 102 others are practicing under the certificates of the board

2

issued after satisfactory examination; and the remaining 703 are non-graduates who have taken out certificates based on years of practice, although not required so to do by law. A number of the 4,780 are also exempt by reason of length of practice before the passage of the act, but have obtained certificates from the board for the purpose of establishing their professional status.

In the first Register, published in June, 1880, there were the names of 5,979 physicians, and in the second, published in December, 1881, 5,987, so that the number of those engaged in medical practice during the last four years shows a slight absolute reduction notwithstanding the increase of population. There has been a gain of 498 graduates and licentiates of medical institutions, these now forming 80 per cent. of the total number, as against 70 per cent. in 1880. The number of licentiates upon examination of the State Board has diminished nearly one-half, chiefly through attendance upon lectures and obtaining diplomas.

During the seven years of its existence, up to the 1st of the present month, the board has issued 8,091 certificates to physicians. Diplomas or licenses have been presented by those now in practice in the State, from 138 medical colleges and licensing bodies in the United States, from ten in Canada, fourteen in Great Britain, thirty-four in Europe, and one each in Africa and South America, making a total of 198. Since the close of the session of 1883, at which time the minimum requirements went into effect, the diplomas of twenty-one colleges in eight different States have been accepted only after an examination of those presenting them in the branches or subjects of the schedule omitted by the respective colleges.

Dr. S. V. Clevenger, well known to our readers as a contributor to these columns, has resigned his position as Pathologist of the Cook County Insane Hospital, and has opened an office for practice in this city, where he proposes to devote his time exclusively to his favorite department in medicine.

---

The Second Annual Meeting of the Ohio State Sanitary Association will be held in Columbus, Ohio, February 5th and 6th, 1885. Programme, containing full information as to papers, session, and railroad rates, will be furnished on application to the Secretary,

R. Harvey Reed, M. D.,
Mansfield, Ohio.

